# Research on Online Social Network Information Leakage-Tracking Algorithm Based on Deep Learning

**DOI:** 10.1155/2022/1926794

**Published:** 2022-06-28

**Authors:** Shuhe Han

**Affiliations:** School of Intelligent Manufacturing and Information, Jiangsu Shipping College, Nantong, Jiangsu 226010, China

## Abstract

The rapid iteration of information technology makes the development of online social networks increasingly rapid, and its corresponding network scale is also increasingly large and complex. The corresponding algorithms to deal with social networks and their corresponding related problems are also increasing. The corresponding privacy protection algorithms such as encryption algorithm, access control strategy algorithm, and differential privacy protection algorithm have been studied and analyzed, but these algorithms do not completely solve the problem of privacy disclosure. Based on this, this article first searches and accurately filters the relevant information and content of online social networks based on the deep convolution neural network algorithm, so as to realize the perception and protection of users' safe content. For the corresponding graphics and data, this article introduces the compressed sensing technology to randomly disturb the corresponding graphics and data. At the level of tracking network information leakage algorithm, this article proposes a network information leakage-tracking algorithm based on digital fingerprint, which mainly uses relevant plug-ins to realize the unique identification processing of users, uses the uniqueness of digital fingerprint to realize the tracking processing of leakers, and formulates the corresponding coding scheme based on the social network topology, and at the same time, the network information leakage-tracking algorithm proposed in this article also has high efficiency in the corresponding digital coding efficiency and scalability. In order to verify the advantages of the online social network information leakage-tracking algorithm based on deep learning, this article compares it with the traditional algorithm. In the experimental part, this article mainly compares the accuracy index, recall index, and performance index. At the corresponding accuracy index level, it can be seen that the maximum improvement of the algorithm proposed in this article is about 10% compared with the traditional algorithm. At the corresponding recall index level, the proposed algorithm is about 5–8% higher than the traditional algorithm. Corresponding to the overall performance index, it improves the performance by about 50% compared with the traditional algorithm. The comparison results show that the proposed algorithm has higher accuracy and the corresponding source tracking is more accurate.

## 1. Introduction

With the rapid development of information technology, especially Internet technology, online social network has increasingly become an important carrier for users to share life and even privacy [1, 2]. With the increasing number and user scale of online social networks, there is a serious risk of disclosure of a large number of user privacy data contained in them. Once the relevant privacy information is leaked, it will cause serious social security problems. Therefore, it is very important and meaningful to protect the personal privacy of relevant users of online social networks [3, 4]. Based on the current situation of online social networks, the main problems are the lack of full understanding of users' trusted identity in the network environment and the lack of recognition mechanism of trusted identity in the current online social networks [5]. Based on this problem, there are many traditional online social network users' personal privacy protection mechanism algorithms. The main algorithms include encryption algorithm, access control strategy algorithm, and differential privacy protection algorithm. However, these algorithms lack corresponding targeted measures at the corresponding leakage tracking level, resulting in many problems in privacy protection tightness, reliability, and corresponding protection efficiency [4, 6]. In order to solve the above problems, the current popular algorithm is mainly to prevent the distribution of relevant information. In order to prevent the leakage of relevant information, this algorithm mechanism adopts the means of “instant messaging and instant deletion” to protect relevant users and monitor illegal means. However, this method is too simple and rough to intelligently solve the above problems [7].

With the continuous development of relevant high-end algorithms such as deep learning algorithms, some columns of algorithms such as deep learning, convolutional neural networks, and reinforcement algorithms continue to appear, which have obvious advantages for solving online social networks with high complexity and large data scale [8]. Conventional deep learning algorithms mainly focus on four levels to track the leaker or protect the user's privacy. Their corresponding mechanisms are mainly the confirmation mechanism based on the user's secret information, on the user's reliable object, on the user's key, and on the user's biological characteristics [9, 10]. Among them, the confirmation mechanism based on user secret information mainly includes user's dynamic password, user name, user password, and corresponding security problems. As the most effective way of this scheme, the corresponding security problems are also the most widely used. The corresponding confirmation mechanism based on user reliability objects mainly includes third-party hardware certificates. The corresponding hardware devices mainly include smart cards, USB, and other hardware devices. Such reliability objects are objects carried by users and can verify their identity [11, 12]. Based on the confirmation mechanism of user key, its main authentication methods include symmetric cryptosystem and asymmetric cryptosystem, and the corresponding representative systems include PKI and other corresponding authentication systems [13]. The confirmation mechanism based on the user's biometrics mainly verifies the user's inherent physiological characteristics and corresponding behavioral characteristics. The main feature mechanism includes face recognition, iris recognition, and digital fingerprint recognition. This method is unique, so its corresponding reliability and accuracy are the highest [14].

Based on the above analysis, this article will solve the problem of the leak-tracking algorithm. Firstly, based on the deep convolution neural network algorithm, this article searches and accurately filters the relevant information and content of online social networks, so as to realize the perception and protection of users' safe content. For the corresponding graphics and data, this article introduces the compressed sensing technology to randomly disturb the corresponding graphics and data. At the level of tracking network information leakage algorithm, this article proposes a network information leakage-tracking algorithm based on digital fingerprint, which mainly uses relevant plug-ins to realize the unique identification processing of users, uses the uniqueness of digital fingerprint to realize the tracking processing of leakers, and formulates the corresponding coding scheme based on the social network topology, and at the same time, the network information leakage-tracking algorithm proposed in this article also has high efficiency in the corresponding digital coding efficiency and scalability. In order to verify the advantages of the online social network information leakage-tracking algorithm based on deep learning, this article compares it with the traditional algorithm. The comparison results show that the algorithm proposed in this article has higher accuracy and the corresponding source tracking is more accurate.

The main sections of the article are arranged as follows: the second section of the article will focus on the research status of online social network information leakage-tracking algorithm based on deep learning. The third section of the article will mainly analyze the optimized online social network information leakage-tracking algorithm and make a specific analysis and research on the information protection algorithm based on the deep convolution neural network algorithm and the network information leakage-tracking algorithm based on digital fingerprint. The fourth section of this article will compare the algorithm proposed in this article with the traditional algorithm and carry out experimental analysis. Finally, this article will be summarized.

## 2. Related Research: Analysis of the Research Status of Online Social Network Information Leakage-Tracking Algorithm Based on Deep Learning

At the corresponding level of individual privacy protection and network information leakage-tracking algorithm, a large number of research institutions and researchers have analyzed it. At the level of individual privacy protection, relevant researchers in the United States have proposed a privacy protection scheme based on k-anonymity, which is one of the most common privacy protection schemes. On this basis, relevant researchers have derived *k*-degree anonymity, *K*-neighbor anonymity, and k-automorphism anonymity. This algorithm is more traditional and has the shortcomings of traditional algorithms [15–18]. In order to solve the disadvantages of the above k-anonymous privacy protection scheme, relevant American researchers further proposed to add a clustering algorithm to the k-anonymous privacy protection scheme in China, which mainly clusters the nodes in the social network into super nodes and uses super nodes to protect the clustered nodes [19,20]. Relevant Japanese researchers have proposed a user privacy protection scheme based on disturbance, which mainly changes the structure of the data by randomly modifying the edges of the chart data owned by the user, so as to protect the user's privacy information. The corresponding random modification mainly includes random increase and decrease and random exchange. Among the corresponding user table data protection methods, relevant European scientific research institutes have proposed a generalized privacy protection strategy, and its main generalization scope includes melon generalization means such as global generalization and subtree generalization [21, 22]. In order to solve the disadvantages of the generalization scheme, relevant Japanese researchers proposed privacy protection based on the division and combination of user data. This algorithm has achieved certain results to a certain extent [23]. For the social network information leakage-tracking algorithm, the current main research directions include the extraction of biometrics and the establishment of the trust model. At the level of corresponding biometric extraction, the mainstream extraction includes biometrics such as digital fingerprint, and the main research details include the research on relevant details such as digital fingerprint decoding, coded fingerprint, continuous fingerprint, and discrete fingerprint [24,25]. For the research on relevant aspects of the trust model, relevant researchers in the United States have proposed a trust model based on network structure, which mainly constructs a trust model based on high-density nodes in the network [26]. Relevant European researchers have proposed a trust model based on user historical interaction, which is mainly constructed by remembering the interaction information between users and past history. Its corresponding evaluation indicators and quantitative indicators include user behavior classification, number of comments, and frequency of user comments. [27,28]. Relevant institutions integrate the advantages of the trust model of network structure and the trust model based on historical interaction, and put forward the concept of the comprehensive model. The trust model built based on the comprehensive model has better modeling advantages to a certain extent. At present, the algorithm mainly focuses on the calculation and construction of the trust model of local non-neighbor users [29–31]. Based on the above research status, it is of obvious value and significance to build an online social network information leakage-tracking algorithm based on deep learning.

## 3. Research and Analysis of Online Social Network Information Leakage-Tracking Algorithm Based on Deep Learning

**Figure 1:**
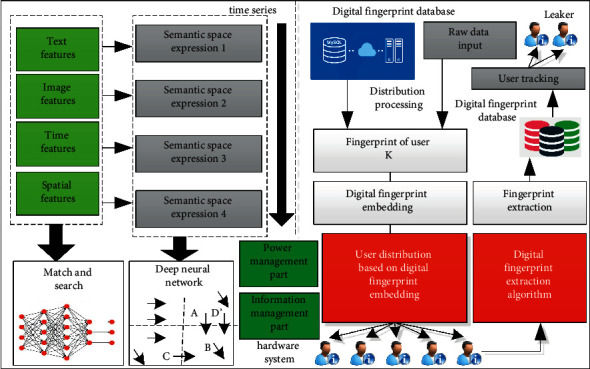
Architecture of online social network information disclosure tracking algorithm based on deep learning.

This section will mainly analyze and study the corresponding core algorithm in this article, that is, the online social network information leakage-tracking algorithm based on deep learning, which mainly includes two core algorithms, namely, the information privacy protection algorithm based on the deep convolution neural network and the network information leakage-tracking algorithm based on digital fingerprint. The corresponding algorithm construction architecture is shown in Figure 1. It can be seen from the figure that the deep convolution neural network model is introduced into the corresponding social network information privacy protection algorithm. Based on this model, firstly, the features of its user-related privacy information and data charts are extracted. The corresponding extracted features include typical features such as text features, image features, time features, and spatial features, and the semantic space is expressed based on these features. In this process, the deep convolution neural network is introduced. In the process of the deep convolution neural network processing, it is necessary to timely introduce the corresponding text into the network and correct its text features, then, process whether the matching between the extracted text and the positive example selection document and the counterexample skip document is consistent, and finally input the corresponding matching results into the deep convolution neural network for processing and analysis. In the process of the corresponding network information leakage-tracking algorithm based on digital fingerprint, it first needs to build the corresponding system model and classify the digital fingerprint based on the social network structure. The corresponding process includes encoding, decoding, and the generation of special coded hash code. The corresponding digital fingerprint generation mainly includes digital fingerprint embedding processing, digital fingerprint distribution, and distribution; the corresponding digital fingerprint extraction and identification process mainly includes the extraction of digital fingerprint and the trigger processing technology of user-related content.

## 4. Analysis and Research on Information Network Privacy Protection Algorithm Based on Deep Convolution Neural Network Algorithm

**Figure 2:**
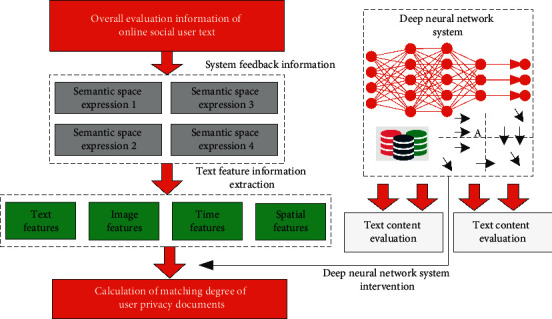
Architecture of the information network privacy protection algorithm based on the deep convolution neural network algorithm.

The algorithm mainly solves the search, extraction, and protection functions of online network privacy corresponding to users. The corresponding algorithm core architecture is shown in Figure 2. The corresponding deep neural network is a technology in the field of machine learning. In supervised learning, the previous multilayer neural network problems are easy to fall into local extremum. If the training samples are enough to completely cover the future samples, the multilayer weight of learning can be well used to predict the new test samples. However, it is difficult to obtain enough labeled samples for many tasks. In this case, simple models, such as linear regression or decision tree, can often get better results (better generalization and worse training error) than multilayer neural networks.

As can be seen from Figure 2, the first is the algorithm search. The corresponding search operations mainly include positive selection, counterexample selection, skip positive example selection, and skip counterexample selection of user privacy text.

After the search of the algorithm, the operation is introduced. Considering the importance of time characteristics, the deep convolution neural network is introduced as an output layer.

Then, it introduces the deep neural network algorithm, which is an important part of this algorithm. When the actual algorithm is running, the reservoir algorithm is used for algorithm processing and analysis, and the deep neural network algorithm is used to calculate the corresponding Q value. Therefore, for a single item, the corresponding reward calculation formula is shown in the following formula:

(1) $$\begin{array}{c} \text{Reo}=\text{ye}-e1{}^{*}\text{ye}-e2{}^{*}\left(\ldots \right){}^{*}\text{ye}-\text{en}{}^{*}, \end{array}$$

where the corresponding *R* represents the result of single text feedback. Based on the above formula, the NCDG feedback calculation formula of the corresponding current text is shown as

(2) $$\begin{array}{c} \text{Reo}=\text{NCDG}\left(r1\right)+\text{NCDG}\left(r2\right)+\cdots \text{NCDG}\left(\text{ri}\right). \end{array}$$

Input the corresponding and processed user privacy text into the deep convolution neural network, and mark the current user text state. The corresponding mark calculation formula is shown as

(3) $$\begin{array}{c} \text{Qe}\left(s,q\right)=\left[y{}^{*}Q1\left(s,a\right)+\cdots y{}^{*}\text{Qe}\left(s,a\right)\right]+r. \end{array}$$

Therefore, the corresponding O-value error calculation formula can be further calculated based on the above formula. The corresponding calculation formula is shown as

(4) $$\begin{array}{c} O=Q\left(s,q\right)-y{}^{*}\max Q\left(s,q\right)-r. \end{array}$$

It can be further seen from the corresponding Figure 2 that the operation strategy of the corresponding information network privacy search and protection algorithm based on the deep convolution neural network is as follows:

  Step 1: input the user's privacy original text into the deep network convolutional neural network, and take it as part of the original input, analyze the characteristics of the current text, and correct the corresponding text characteristics.
  Step 2: calculate the matching degree between the current user's privacy document and the current positive example text and the skipped counterexample text, and compare the relative values of the two. If the score value of the corresponding counterexample text is high, return to step 1 for processing. When the score value of the corresponding positive example is high, continue the algorithm.
  Step 3: Input the corresponding text feature matching results into the long-term and short-term neural networks in the deep neural network, and enter the additional time features into the system as gate input, so as to further strengthen the enhancement effect of text features.
  Step 4: input the corresponding fusion results into the deep neural network reinforcement learning to obtain the reinforcement score value of the corresponding neural network, and add the corresponding single user document to the document sequence to calculate the score value of the whole system.
  Step 5: take the single document of the corresponding user as the score feedback value, input it into the reinforcement learning neural network, and input the total score of the current text network into the reinforcement learning network for analysis.
  Step 6: repeat steps 1–5 to process all the data of the user.

## 5. Research and Analysis of Network Information Leakage-Tracking Algorithm Based on Digital Fingerprint

**Figure 3:**
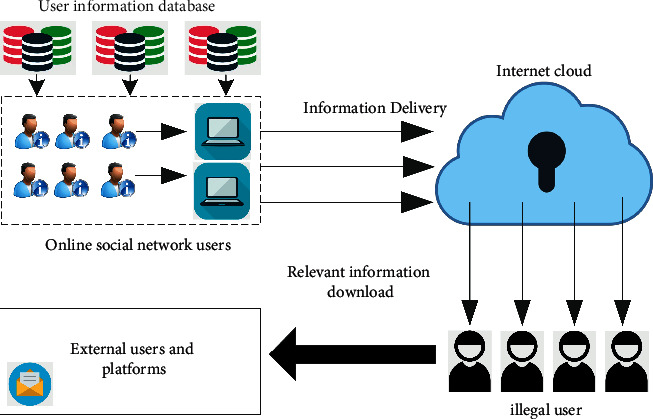
Framework diagram of personal information disclosure model of online social network users.

The typical framework diagram of the information disclosure model is shown in Figure 3. It can be seen from the figure that most scenarios are when users publish the corresponding personal privacy information to the network, the corresponding privacy will be downloaded by other users, resulting in the corresponding personal privacy disclosure.

When designing the network information leakage-tracking algorithm, this article is mainly based on the digital fingerprint. The analysis of its characteristics in the social network mainly focuses on the following characteristics: it cannot provide unique digital fingerprint for hundreds of millions of users in essence, the digital fingerprint in the corresponding social network is dynamic, and the digital fingerprint in the corresponding social network does not involve the corresponding conflict of law and interests.

**Figure 4:**
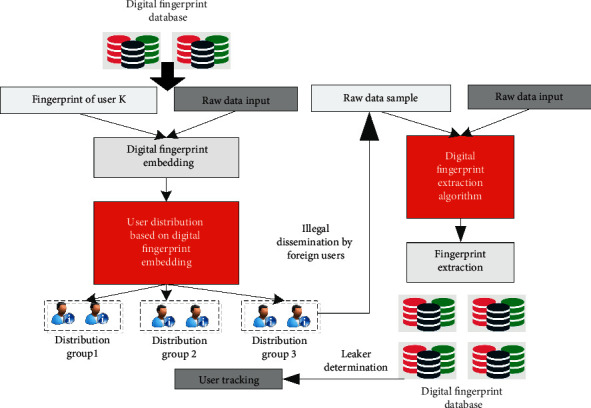
Digital fingerprint system model block diagram of the online social network.

Based on this, the system model of digital fingerprint scheme is established. The corresponding digital fingerprint system model is shown in Figure 4. The corresponding system model should include three modules: digital fingerprint coding algorithm module, digital fingerprint embedding algorithm module, and digital fingerprint detection algorithm module. In the actual algorithm processing, we first need to group a large number of online network user data. The traditional orthogonal code is not suitable for online social networks. Based on this, this article uses hash code as coding. In the hash code generation step, firstly, the unsupervised learning method is used to train the corresponding data set and obtain the classifier vector with the same length as the hash code. Then, the support vector machine model is used to classify the user data for many times and integrate the data calculation results.

**Figure 5:**
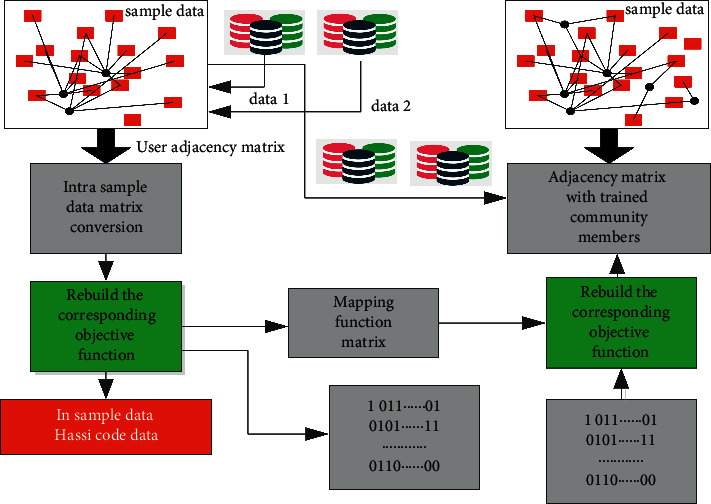
Digital fingerprint generation index diagram of the online social network.

The generation framework of the corresponding hash code and the corresponding mapping function is shown in Figure 5. From the figure, we can further draw the corresponding generation steps as follows:

  Step 1: based on the local preserving projection algorithm, a local or global projection preserving algorithm for system data is proposed.
  Step 2: introduce the concept of matrix transformation, maintain the nearest neighbor characteristics of the original spatial structure of the data, and reconstruct the corresponding objective function.
  Step 3: transform the corresponding objective function into Hilbert space for processing and analysis.
  Step 4: select the corresponding data samples as the boundary and mark them, carry out corresponding training on the corresponding marks, and use the corresponding machine model to predict and process the remaining samples.
  Step5: use the *k*-means algorithm to obtain the cluster center as the mark, use the sample data in the mark to generate the hash code, and train the mapping matrix. The corresponding training matrix is the trained mapping matrix.
  Step 6: introduce corresponding positive selection items to avoid over fitting during training.

Based on the above hash code and the corresponding binary random sequence code, the user's unique digital fingerprint is formed. The whole digital fingerprint analysis process is as follows: continuously input the user's online social network adjacency matrix. Determine the user online network training dataset based on the training center. Generate the corresponding kernel data matrix K; using the corresponding algorithm to generate hash code and corresponding mapping function; generating a new hash code for the user data outside the sample through the mapping function; classify the corresponding communities based on the hash code; generate binary sequence code for the corresponding community based on the classified community. The digital fingerprint of online network users is generated based on the generated hash code and binary sequence code, and the corresponding index is generated based on the digital fingerprint.

Based on the above regeneration and definition of the user's digital fingerprint, the unique mark of the user's information characteristics can be realized. Through the unique mark, the tracking processing of the relevant personnel who steal the user's information can be realized, and then, the accurate tracking of the information disclosure personnel can be completed.

## 6. Algorithm Comparison Experiment and Analysis

**Figure 6:**
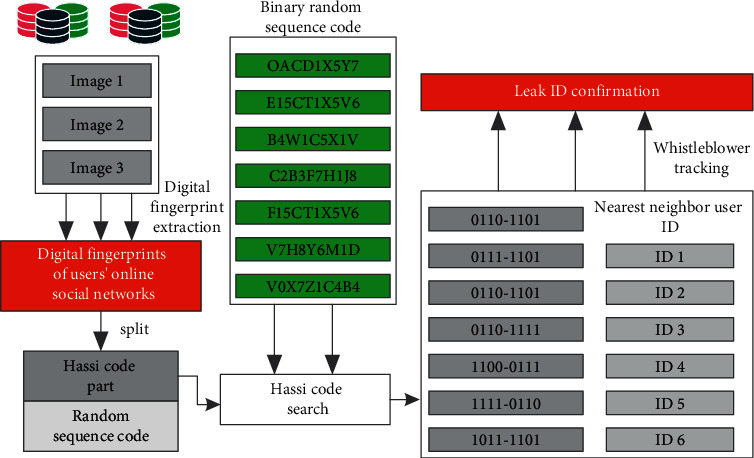
Verification flowchart of the information network privacy protection algorithm based on the deep convolution neural network algorithm.

In order to verify the superiority of the algorithm in this article, the experimental verification is carried out. The corresponding verification flowchart is shown in Figure 6. It can be seen from the figure that firstly, the user information is extracted based on the algorithm in this article and the traditional algorithm, and the digital fingerprint processing mark is carried out according to its corresponding characteristics. The corresponding digital fingerprint is split to split the hash code and random sequence code of the user's privacy information. The corresponding hash code is indexed, and the corresponding random sequence code is processed with its corresponding digital fingerprint and the digital ID card of the corresponding adjacent user, so as to calculate the digital ID of the corresponding leaky user, so as to realize the tracking of the leaker.

The experiment mainly follows the control variable method, and the verified indicators include accuracy test indicators, recall indicators, and overall system performance indicators.

*Accuracy test index*: during the recall index test and analysis, the sample environment tested by the control system is consistent and the test data attack type is consistent.

*Recall index*: during the recall index test, the sample environment tested by the same control system is consistent, and the type of test data attack is consistent. Mainly check the recall.

*Overall performance index of the system*: the overall performance index of the system is mainly standardized and comprehensively evaluated through various indexes, so as to obtain a systematic data index evaluation.

**Figure 7:**
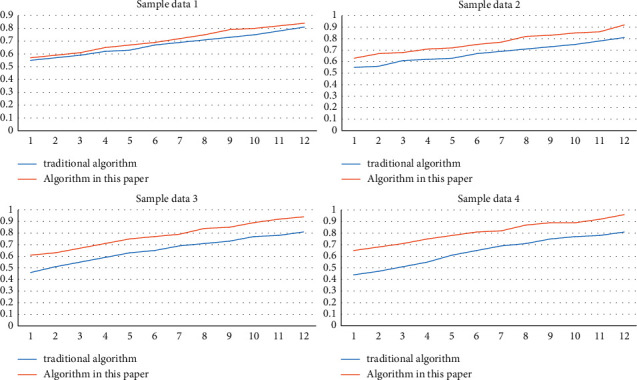
Network structure accuracy test curve.

In order to verify the accuracy of the network structure of the algorithm in this article, the experiment is carried out based on three data packets, and the corresponding numbers of data packet nodes are 40238045 and 10023. The cross-verification experiment is carried out on the above data packets. The verification tool used is MATLAB, and the corresponding network structure accuracy test curve is shown in Figure 7. As can be seen from Figure 7, with the increase in the hash code data length, the corresponding algorithm proposed in this article has obvious advantages in the query accuracy of the nearest neighbor set compared with traditional algorithms. By analyzing the specific reasons, we can see that with the continuous increase of the length of hash code, the probability of mapping its corresponding nonadjacent data set into hash code will continue to decrease, and the corresponding distance will continue to increase. Therefore, the less likely it is to be judged as a nearest neighbor dataset.

**Figure 8:**
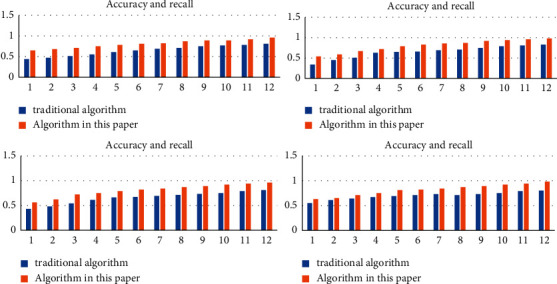
Accuracy and recall curve of information network privacy protection algorithm based on the deep convolution neural network algorithm.

In order to further verify the accuracy of this algorithm and the traditional algorithm in the digital fingerprint scene, this article embeds and extracts the digital fingerprint of the real digital image. The corresponding accuracy rate and recall rate curve are shown in Figure 8. It can be seen from the figure that the value of the corresponding accuracy rate decreases with the increasing value of the recall rate. At the same time, this feature is relatively obvious in this algorithm. When the corresponding recall rate is high, the corresponding accuracy rate is relatively low, and when the corresponding recall rate is low, the corresponding accuracy rate is relatively high. Based on this, we can find the nearest neighbor set of hash code. Based on this nearest neighbor set, we can compare the distance between the corresponding target fingerprint and the nearest neighbor set fingerprint according to the binary sequence code, so as to further determine the ID information of the leaker.

**Figure 9:**
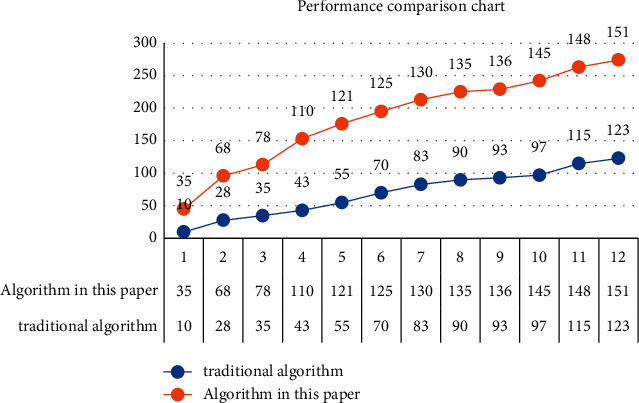
Performance comparison between information network privacy protection algorithm based on the deep convolution neural network algorithm and traditional algorithm.

In order to further verify the performance advantages of the digital fingerprint leak tracking algorithm proposed in this article compared with the traditional digital fingerprint algorithm, the performance test experiment is carried out in this article. The corresponding experimental data come from an authoritative database. The experimental data contains 1000–1000 data nodes, and the corresponding number of experiments is set to 10. Based on this experiment, first set the corresponding distance value to 4, and the corresponding hash code and binary random sequence code to 64 bits. The corresponding experimental results are shown in Figure 9. From the figure, it can be seen that the optimized digital fingerprint tracking algorithm proposed in this article has obvious advantages in system performance compared with the traditional digital fingerprint algorithm. Obviously, with the continuous rise of data nodes, the performance gap between the corresponding algorithm in this article and the traditional algorithm will be larger and larger, and the corresponding performance advantages will be more and more obvious. The specific reasons are as follows: in this article, the digital fingerprint index table sorted based on hash code is preset. When the actual algorithm runs, it first searches and processes based on the adjacent hash code, then searches and analyzes in the corresponding adjacent set based on the binary random sequence code, and finally determines the corresponding ID based on the minimum distance and locks the ID information of the leaker.

Based on the above analysis, compared with the traditional algorithm, the algorithm proposed in this article has obvious advantages in the accuracy of network structure, the accuracy of digital fingerprint, and the performance of digital fingerprint. It has obvious significance to further improve the determination efficiency of relevant information of online social leakers.

## 7. Conclusion

This article mainly analyzes and studies the problems faced by the current development of online social networks, which systematically analyzes and studies the research status of network information leakage-tracking algorithm and deep learning algorithm, and points out the possibility and necessity of the integration of the two algorithms. Based on this background, this article searches and accurately filters the relevant information and content of online social networks based on the deep convolution neural network algorithm, so as to realize the perception and protection of users' safe content. For the corresponding graphics and data, this article introduces the compressed sensing technology to randomly disturb the corresponding graphics and data. At the level of tracking network information leakage algorithm, this article proposes a network information leakage-tracking algorithm based on digital fingerprint, which mainly uses relevant plug-ins to realize the unique identification processing of users, uses the uniqueness of digital fingerprint to realize the tracking processing of leakers, and formulates the corresponding coding scheme based on the social network topology, and at the same time, the network information leakage-tracking algorithm proposed in this article also has high efficiency in the corresponding digital coding efficiency and scalability. In order to verify the advantages of the online social network information leakage-tracking algorithm based on deep learning, this article compares it with the traditional algorithm. In the experimental part, this article mainly compares the accuracy index, recall index, and performance index. At the corresponding accuracy index level, it can be seen that the maximum improvement of the algorithm proposed in this article is about 10% compared with the traditional algorithm. At the corresponding recall index level, the algorithm proposed in this article is about 5–8% higher than the traditional algorithm. Corresponding to the overall performance index, the algorithm proposed in this article improves the performance by about 50% compared with the traditional algorithm. The comparison results show that the algorithm proposed in this article has higher accuracy and the corresponding source tracking is more accurate. In the follow-up research, this article will focus on the defect of high load of the algorithm proposed in this article and optimize the algorithm based on this defect, so as to further improve the accuracy and reliability. At the same time, this article will also carry out in-depth research on the relevant theories of deep learning and better combine it with the online social network leak tracking algorithm to improve the performance of the algorithm.

## Data Availability

The data used to support the findings of this study are available from the corresponding author upon request.
